# Sir Richard Quain

**Published:** 1898-04

**Authors:** 


Sir Richard Quain, Bart., physician extraordinary to her Majesty the Queen, president of the General Medical Council, and editor of the *Dictionary of Medicine*, died March 13, 1898, aged 81 years. He was born October 30, 1816, was a fellow of several learned societies, and the author of numerous medical and scientific works.

